# An evidence-based warfarin management protocol reduces surgical delay in hip fracture patients

**DOI:** 10.1007/s10195-013-0274-7

**Published:** 2013-11-26

**Authors:** I. Ahmed, M. A. Khan, V. Nayak, A. Mohsen

**Affiliations:** 1 Department of Anaesthesia and Critical Care, Hull Royal Infirmary, Anlaby Road, Kingston upon Hull, HU3 2JZ UK; 2 CT2 Surgery, Hull Royal Infirmary, Kingston upon Hull, UK; 3 Department of Anaesthesia, Hull Royal Infirmary, Kingston upon Hull, UK; 4 Department of Trauma & Orthopedics, Kingston upon Hull, UK

**Keywords:** Warfarin, Anticoagulation, Trauma surgery, Hip fracture, Vitamin K, Protocol, Elderly

## Abstract

**Background:**

Up to 4 % of patients presenting with a hip fracture may be on warfarin at admission. There is little consensus on the timing, dosage or route of vitamin K administration. We aimed to evaluate the impact of a locally developed, evidence-based protocol for perioperative warfarin management on the admission-to-operation time (AOT) in hip fracture patients.

**Materials and methods:**

Clinical and demographic data were collected prospectively for hip fracture patients who were on warfarin at the time of admission (post-protocol group) and compared to a historical control group of patients who were on warfarin before implementation of the protocol (pre-protocol group). Univariate analysis was undertaken to identify any significant differences between the two groups.

**Results:**

Twenty-seven patients in the pre-protocol group (27/616, 4.4 %) and 40 patients in the post-protocol group (4.7 %, 40/855) were on warfarin at admission. There was a significant reduction in the median AOT from 73 h (IQR 46–105) to 37.7 h (IQR 28–45) after implementation of the warfarin protocol (*p* < 0.001). The proportion of patients operated on within 48 h of admission increased from 30 % (8/27) in the pre-protocol group to 80 % (32/40) in the post-protocol group (*p* < 0.001). No significant differences in hospital length of stay (*p* = 0.77) or the postoperative warfarin recommencement time (*p* = 0.90) were noted between the two groups.

**Conclusion:**

Implementation of a perioperative warfarin management protocol can expedite surgery in hip fracture patients, but did not reduce hospital stay in our cohort, possibly because of a delay in recommencing warfarin in these patients postoperatively.

**Level of evidence:**

Level III.

## Introduction

Timely preoperative optimisation of hip fracture patients is critical in allowing urgent surgery [1–4]. Delays in surgical management are associated with poor outcomes. It is estimated that up to 4 % of these patients are on an anticoagulants such as warfarin for a variety of medical conditions [5, 6]. The British Society of Haematology recommends preoperative discontinuation of warfarin and administration of vitamin K in order to expedite INR reduction in these patients [7]. An international normalised ratio (INR) of <1.5 is required before hip surgery can proceed safely [4]. Re-warfarinisation is recommended on the first postoperative day, provided adequate haemostasis has been achieved during surgery [7]. Existing guidelines on vitamin K administration are ambiguous. As a result there is wide variation in clinical practice across the UK [8] with little or no consensus on the timing, dosage or route of administration of vitamin K prior to hip fracture surgery or recommencement of warfarin post-surgery [9–12]. We hypothesised that implementation of a locally developed, evidence based protocol for perioperative warfarin reversal and postoperative re-warfarinisation would significantly reduce the admission-to-operation time (AOT) in hip fracture patients. We also aimed to determine if implementation of such a protocol would lead to a reduction in the postoperative warfarin recommencement time and overall length of stay in hospital.

## Materials and methods

The study was approved by the clinical governance team at our institution. It was confirmed that the project fulfilled the criteria of a clinical audit as defined in the NHS National Research Ethics Service document entitled ‘Defining Research’ [13] and formal ethical approval from the NHS research and ethics committee was therefore not deemed necessary. The study was performed in accordance with the ethical standards of the 1964 Declaration of Helsinki as revised in 2000. Since the study involved evaluation of the routine clinical care received by hip fracture patients, formal consent from individual patients was not warranted, and was waived by the clinical governance team. However, implied consent was sought through information leaflets by making all patients aware of continued clinical auditing to improve clinical care received by patients.

**Fig. 1:**
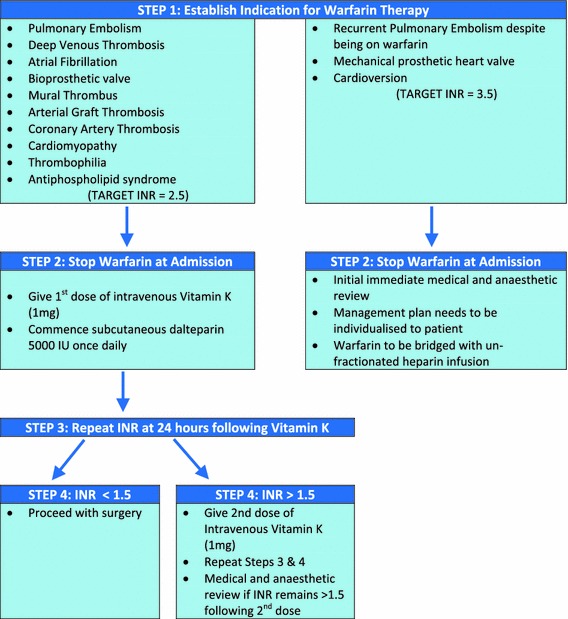
Algorithm for reversal of warfarin therapy in hip fracture patients

**Fig. 2:**
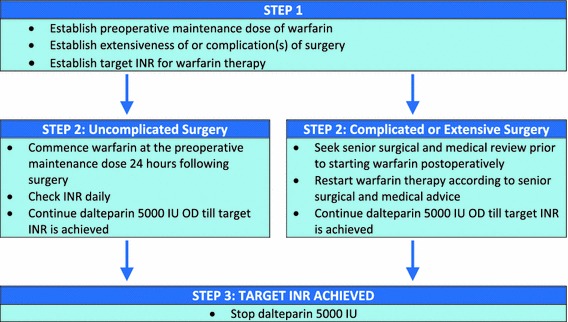
Algorithm for reintroduction of warfarin following surgery

A retrospective case series evaluation conducted at our institution in July 2010 showed a significant delay in the operative fixation of hip fractures in patients on warfarin therapy at the time of admission. This led to a multidisciplinary consultation involving orthopaedic surgeons, haematologists and medical and nursing health staff with subsequent development of a local protocol for warfarin management in hip fracture patients. This protocol was based on guidance issued by the British Society of Haematology [7] and American Heart Association [11] and adopted an algorithm-based approach towards preoperative warfarin discontinuation (Fig. 1) and postoperative recommencement of warfarin in hip fracture patients (Fig. 2). The protocol was implemented at our institution in August 2010. The prospective arm of the study directly followed the historic case series. All patients with an INR ≤1.5 were operated on the next available trauma list. We hypothesised that implementation of a locally developed perioperative warfarin management protocol would lead to a significant reduction in the AOT for hip fracture patients on warfarin therapy at admission. We defined AOT as the interval between admission of a patient into the emergency department or diagnosis of fracture if the patient was already in hospital (in-patient injury) and start of induction of anaesthesia.

All hip-fracture patients that underwent hip fracture surgery at our institution were identified for inclusion into the study from our locally developed ‘hospital hip-fracture database’. Demographic, clinical and surgery-related data were obtained from patient case notes and the in-house electronic patient administration system (iSOFT 2010). The retrospective arm of the study extended from 1st April 2009 to 30th June 2010 and included 616 consecutive patients of whom 27 were on warfarin at the time of admission (pre-protocol group). The prospective arm extended from 1st September 2010 to 31st December 2012 and included 855 consecutive hip fracture patients, of whom 40 were on warfarin at admission. Patients admitted during the months of July and August 2010 (*n* = 97) were excluded from the analysis to allow development and implementation of the warfarin protocol.

Demographic data collected for all patients included age, gender and preoperative residential status. Clinical data, including ASA (American Society of Anaesthesiology) grade, indication for anticoagulation, INR before and after surgery and prior to discharge, pre- and postoperative haemoglobin (Hb) levels, time interval from admission to vitamin K administration, time interval from vitamin K administration to operation and from operation to recommencement of warfarin were collected on all patients. Any adverse effects of vitamin K administration, including allergic reactions or thromboembolic complications were noted. Pre- and postoperative Hb levels were measured immediately prior to surgery and at 24 h following surgery, respectively and the peri-procedural fall in Hb was calculated by subtracting the postoperative Hb level from the preoperative Hb level.

Surgery related data, including the type of implant used, grade of operating surgeon/anaesthetist, the type of anaesthetic technique used and hospital length of stay were also collected. Data on the grade of surgeon and anaesthetist was categorised into two groups: consultant grade or other (including all types of trainees and staff grade doctors). The type of anaesthetic technique was also categorised into two groups: general anaesthesia with or without a nerve block (GA) and regional anaesthesia including spinal, epidural or combined spinal-epidural anaesthetic (RA). Statistical analysis was undertaken to compare differences between the two groups.

Descriptive statistics of median (interquartile range, IQR) and mean (standard deviation, SD) were calculated for continuous variables, whereas proportions as percentages were used for categorical variables. The Mann–Whitney (MW) test was used to compare continuous variables whereas Fisher’s exact (FE) and Chi-square (CS) tests were used to compare categorical variables on univariate analysis. All statistical analyses were performed using XLSTAT version 7.0 software (Addinsoft, New York, NY, USA).

## Results

Twenty-seven patients in the pre-protocol group (27/616, 4.4 %) and 40 patients in the post-protocol group (4.7 %, 40/855) were on warfarin at admission. The demographic, clinical and surgery-related characteristics of patients in both groups are presented in Table 1. No significant differences in age (*p* = 0.67), gender (*p* = 1.00), preoperative residential status (*p* = 0.76), ASA grade (*p* = 0.06), type of implant used (*p* = 0.85) grade of operating surgeon (*p* = 0.80) or anaesthetist (*p* = 0.40) and type of anaesthetic technique used (*p* = 0.80) were noted between the two groups. There was a significant difference in the indications for anticoagulation therapy between the two groups with a higher proportion of patients in the post-protocol group noted to have atrial fibrillation compared to the pre-protocol group (34 vs. 16 respectively, *p* = 0.001). The mean INR levels recorded at admission, preoperatively and at discharge in both groups are shown in Table 1; no significant differences in INR levels were noted between the two groups. No significant difference in the peri-procedural fall in Hb was identified between the two groups (mean fall in Hb was 1.9 g/dl (SD 1.1) in the pre-protocol group vs. 2.3 g/dl (SD 1.2) in the post-protocol group, *p* = 0.34).

**Table 1:** Demographic, clinical and surgery-related characteristics of all patients

Variable	Pre-protocol (*n* = 27)	Post-protocol (*n* = 40)	*p* value
Age, median (IQR)	81 (77–85)	81.5 (76–88)	0.67^‡^
Gender			
Male	9 (33 %)	18 (45 %)	1.0*
Female	14 (67 %)	26 (55 %)	
Residential status			
Own home	19 (70.3 %)	30 (75 %)	0.76^†^
Residential home	6 (22.2 %)	6 (15 %)	
Nursing home	2 (7.4 %)	3 (7.5 %)	
In-patient	0 (0 %)	1 (2.5 %)	
ASA grade			
I	0 (0 %)	0 (0 %)	0.06^†^
II	12 (44.4 %)	7 (17.5 %)	
III	9 (33 %)	19 (47.5 %)	
IV	6 (22.2 %)	14 (35 %)	
Indication for warfarin therapy			
Atrial fibrillation	16 (59.3 %)	34 (85 %)	0.001^†^
Pulmonary embolism	6 (22.2 %)	3 (7.5 %)	
Deep venous thrombosis	3 (11.1 %)	2 (5 %)	
Others (thrombocytosis/valve replacement)	2 (7.4 %)	1 (2.5 %)	
INR, mean (SD)			
At Admission	3.4 (3.6)	3.3 (2.6)	0.08^‡^
Preoperative	1.2 (0.2)	1.3 (0.2)	0.05^‡^
At Discharge	2.3 (0.7)	2.4 (0.8)	0.73^‡^
Hb level (g/dl), mean (SD)			
Preoperative Hb	12.3 (1.5)	12.9 (1.9)	0.12^‡^
Postoperative Hb	10.3 (0.3)	10.6 (0.3)	0.57^‡^
Fall in Hb	1.9 (1.1)	2.3 (1.2)	0.34^‡^
Type of implant			
Hemiarthroplasty	13 (48.1 %)	20 (50 %)	0.85^†^
DHS/CHS	11 (40.7 %)	14 (35 %)	
Intra-medullary fixation	3 (11.1 %)	6 (15 %)	
Surgeon grade			
Consultant	11 (40.7 %)	15 (37.5 %)	0.80*
Other	16 (59.3 %)	25 (62.5 %)	
Anaesthetist grade			
Consultant	26 (96.3 %)	40 (100 %)	0.40*
Other	1 (3.7 %)	0 (0 %)	
Anaesthesia technique			
General	14 (51.8 %)	23 (57.5 %)	0.80*
Regional	13 (48.2 %)	17 (42.5 %)	

*IQR* interquartile range, *SD* standard deviation, *Hb* haemoglobin, *INR* international normalised ratio, *DHS* dynamic hip screw, *CHS* cannulated hip screw

* Fisher’s exact test

^†^Chi-square test

^‡^Mann–Whitney test

There was a significant reduction in the median AOT (*p* < 0.001) from 73 h (IQR 46–105) in the pre-protocol group to 37.7 h (IQR 28–45) in the post-protocol group (Table 2). Both the admission to vitamin K administration time [18 h (IQR 8–24) in pre-protocol group vs. 7.6 h (IQR 4–13) in post-protocol group, *p* = 0.003] and the vitamin K administration to operation time [64 h (IQR 24–75) in pre-protocol group vs. 29.3 h (IQR 17–37) in post-protocol group, *p* = 0.005], reduced significantly after implementation of the protocol. The proportion of patients operated on within 48 h of admission increased from 30 % (8/27) in the pre-protocol group to 80 % (32/40) in the post-protocol group (*p* < 0.001). A surgical delay of more than 48 h due to a prolonged INR (>1.5) occurred in 14 (52 %) patients in the pre-protocol group compared to 5 (12.5 %) patients in the post-protocol group.

**Table 2:** Effect of protocol implementation on route of vitamin K administration and outcomes

Variable	Pre-protocol (*n* = 27)	Post-protocol (*n* = 40)	*p* value
Route of VK administration			
Intravenous	12 (44.4 %)	36 (90 %)	<0.001^†^
Oral	3 (11.1 %)	0 (0 %)	
None	12 (44.4 %)	4 (10 %)	
Admission to VK administration time (h), median (IQR)	18 (8–24)	7.6 (4–13)	0.003^‡^
VK administration to operation time (h), median (IQR)	64 (24–75)	29.3 (17–37)	0.005^‡^
Operation to warfarin recommencement time (h), median (IQR)	68.0 (50–100)	73.8 (51–100)	0.90^‡^
Admission to operation time (h), median (IQR)	73 (46–105)	37.7 (28–45)	<0.001^‡^
Surgery within 48 h of presentation	8 (30 %)	32 (80 %)	<0.001^†^
Hospital length of stay	21.2 (16–36)	19.2 (14–33)	0.77^‡^

*IQR* interquartile range, *VK* vitamin K

^†^Chi-square test

^‡^Mann–Whitney test

The warfarin recommencement time following surgery in the pre-protocol group was 68 h (IQR 25–100) compared to 73.8 h (IQR 51–100) in the post-protocol group (*p* = 0.90). The overall median length of hospital stay decreased from 21.2 days (IQR 6–35) in the pre-protocol group to 19.2 days (IQR 14–33) in the post-protocol group (*p* = 0.77). These differences were not statistically significant.

In the pre-protocol group, vitamin K was administered for warfarin reversal in 15 (55.6 %) patients preoperatively (12 received intravenous vitamin K whereas 3 received oral vitamin K). Of these 15 patients that received vitamin K, 6 required a second dose to correct the INR to <1.5. In comparison, 36 (90 %) patients in the post-protocol group received intravenous vitamin K for warfarin reversal preoperatively; 21 required a second dose and another 6 required a third dose for correction of INR prior to surgery. Of the four patients that did not receive vitamin K in the post-protocol group, three had an INR of <1.5 at admission and one had an elevated INR of 17.5 which was treated with prothrombin concentrate complex (PCC). No adverse reactions to vitamin K administration were noted in either of the two groups. There were no thromboembolic events recorded for any of the patients in either group at 3 months follow-up.

## Discussion

Our results show that implementation of a locally developed perioperative anticoagulation management protocol utilising intravenous vitamin K significantly reduced admission to vitamin K administration time and vitamin K administration to operation time in hip fracture patients who were on warfarin therapy at the time of admission. As a result, hip fracture surgery at our institution was expedited and significant reductions in the admission to operation time were seen. Delays in surgery due to a prolonged INR (>1.5) were also reduced. Use of the protocol did not appear to reduce overall hospital stay in our cohort or the time taken for recommencing warfarin postoperatively. Perioperative administration of intravenous Vitamin K was not associated with any thromboembolic or allergic complications.

There has been an exponential increase in the number of patients taking warfarin over the last two decades. This increase can partly be attributed to a rise in the prevalence of atrial fibrillation due to an ageing population and partly to improvements in the diagnosis and management of patients with arrhythmias in primary care. We found the overall prevalence of warfarin use to be 4.6 % (67/1,471) in our cohort of hip fracture patients.

There is growing evidence that provision of protocol- or checklist-driven standardised care can improve clinical outcomes [14–16]. Ashouri et al. [6] demonstrated that lack of a standardised protocol resulted in variations in the anticoagulation reversal regime and consequently lead to delays in hip fracture surgery. The Scottish National audit showed that the proportion of medically fit hip-fracture patients operated on within 24 h of admission rose from 86 to 97 %, following introduction of protocols to standardise management [17]. Our results demonstrate that the proportion of patients that underwent surgery within 48 h of presentation increased from 30 % before the existence of a standardised protocol to 80 % after its implementation.

Simply waiting for the INR to fall to acceptable levels in hip fracture patients who are on warfarin may lead to significant delays in surgery [5, 6, 14, 18–20]. There is a clear need for interventions to reduce the INR urgently in these patients, however, no consensus exists on the timing, route or dosage of anticoagulation reversal. UK guidance on perioperative management of hip-fracture patients on warfarin therapy is confusing at worst and non-conclusive at best. The British Orthopaedic Association (BOA) recommends that the INR should be corrected to 1.5 or lower prior to surgery [4] but does not support the use of vitamin K or fresh frozen plasma (FFP) due to a lack of high-level evidence. Guidelines published by the British Committee for Standards in Haematology (BCSH) do not make specific references to the perioperative management of warfarin in hip fracture patients [7]. Although they appear to support warfarin reversal with intravenous vitamin K for urgent surgery, the safe or effective dose of vitamin K is not specified. The Scottish Intercollegiate Guidelines Network (SIGN) guidelines suggest that orally administered low-dose vitamin K (1–2.5 mg) can take up to 24 h to reverse the effects of warfarin [2]. They recommend that the route and dose of vitamin K should depend on the INR at admission and urgency of surgery. Our study has shown that the use of intravenous vitamin K at doses of 1 mg administered at 24-h intervals in low risk patients can reduce surgical delay in hip-fracture patients safely. Furthermore, we did not find any significant adverse reactions to intravenous vitamin K or an increase in thromboembolic events at 3 months post-surgery.

Development of the protocol utilised in the current study was driven by two factors: the existence of uncertainty in the perioperative management of hip fracture patients on warfarin therapy due to non-specific national guidelines and a local retrospective audit that showed suboptimal management of hip-fracture patients because of wide variations in management by clinicians; some choosing the ‘wait and watch’ approach to correct INR, some using oral vitamin K while others used intravenous vitamin K for warfarin reversal. Our protocol was influenced by BCSH recommendations; however, three distinctive features can be identified:

1. Thromboembolic risk stratification of patients preoperatively into two groups (low and high risk) with different action triggers for warfarin discontinuation, reversal and bridging.
2. Use of a standardised 1-mg dose of intravenous vitamin K.
3. Bleeding risk stratification of patients postoperatively into two groups (simple-uncomplicated and extensive-complicated) with separate action triggers for re-warfarinisation.

The primary concern regarding discontinuation of warfarin and its reversal with vitamin K is the predisposition of patients to thromboembolic events such as stroke, deep vein thrombosis, pulmonary embolism and prosthetic heart valve thrombus formation. It is estimated that in high-risk patients, the absolute risk of thromboembolic events within 6–8 days of stopping warfarin is approximately 0.3 % [21]. We classified patients on warfarin into low- and high-risk groups depending on the indication for warfarin therapy, and linked action triggers for vitamin K administration to low risk patients only. High-risk patients were managed after multidisciplinary discussion between surgeons, anaesthetists and haematologists. Only one high-risk patient was identified in our study. We believe that withholding vitamin K in high-risk patients does not represent suboptimal care. It reflects a safe approach to managing these patients in whom valve thrombosis can be a devastating complication [22].

Five different approaches to correction of INR have been proposed; *rapid* with PCC, *fast* with FFP, *prompt* with intravenous vitamin K, *slow* with oral vitamin K and *ultra*-*slow* with simple discontinuation of warfarin [23]. We did not consider the first two approaches because of significant safety concerns associated with use of blood-derived products and the non-emergency nature of hip fracture surgery. Although simply discontinuing warfarin can eventually lead to a drop in INR, this may take up to 5 days [24]. Both the oral and intravenous routes are considered equally effective for vitamin K administration but the oral route has been shown to be slower for INR correction and can delay surgery by more than 24 h [25]. It is for these reasons that we chose the intravenous route for our protocol. We chose a 1-mg dose of vitamin K for warfarin reversal. Although higher doses could potentially offer faster INR correction, they also confer increased warfarin resistance, making re-warfarinisation more difficult postoperatively [11, 26].

Warfarin could be recommenced safely within 24 h following surgery provided adequate haemostasis had been achieved [7]. Despite implementation of the protocol, we did not see a significant difference in the postoperative warfarin recommencement time. This could be attributed to inconsistencies in application of the protocol by clinical staff prescribing warfarin on the wards and has been highlighted to the orthogeriatric and ward-based surgical teams. We hypothesise that a possible reason for reluctance among clinicians to recommence warfarin within 24 h after surgery is a lack of awareness on intraoperative haemostasis. We have therefore asked surgeons to clearly document satisfaction with haemostasis in operative case notes and include orders to restart warfarin within 24 h in the postoperative plan. Although we noted a 2-day reduction in median length of stay in the post-protocol group, this decrease was not found to be statistically significant. We believe that further reductions may well be seen if strict adherence to the postoperative warfarin recommencement regimen could be ensured, however, we do recognise that length of stay in hip fracture surgery patients is multifactorial.

Our study is limited by its observational nature and the use of a retrospective series of patients as the control group. We recognise that there were significant differences between the pre- and post-protocol groups with respect to the indications for warfarin, however, patients in both groups were well matched for age, gender and ASA grade. We conclude that variations in the management of hip fracture patients on warfarin therapy at admission could be reduced by introduction of a simple, evidence-based protocol, and that implementation of such a standardised tool using intravenous vitamin K is a safe and effective method to expedite hip fracture surgery and prevent delays.
